# The Experience of Caregivers Living with Cancer Patients: A Systematic Review and Meta-Synthesis

**DOI:** 10.3390/jpm5040406

**Published:** 2015-11-19

**Authors:** Peeranuch LeSeure, Supaporn Chongkham-ang

**Affiliations:** McCormick Faculty of Nursing, Payap University, Chiang Mai 50000, Thailand; E-Mail: Goodmorningpim@hotmail.com

**Keywords:** caregiver, cancer, experience, systematic review, meta-synthesis

## Abstract

The objectives of this meta-synthesis were to: (1) explore the experience of caregivers who were caring for cancer patients, including their perceptions and responses to the situation; and (2) describe the context and the phenomena relevant to the experience. Five databases were used: CINAHL, MEDLINE, Academic Search, Science Direct, and a Thai database known as the Thai Library Integrated System (ThaiLIS). Three sets of the context of the experience and the phenomena relevant to the experience were described. The contexts were (1) having a hard time dealing with emotional devastation; (2) knowing that the caregiving job was laborious; and (3) knowing that I was not alone. The phenomenon showed the progress of the caregivers’ thoughts and actions. A general phenomenon of the experience—balancing my emotion—applied to most of the caregivers; whereas, more specific phenomenon—keeping life as normal as possible and lifting life above the illness—were experienced by a lesser number of the caregivers. This review added a more thorough explanation of the issues involved in caregiving for cancer patients. A more comprehensive description of the experience of caregiving was described. The findings of this review can be used to guide clinical practice and policy formation in cancer patient care.

## 1. Introduction

Cancer and cancer treatment affect not only the patients, but also their family members and caregivers. When giving care to persons with life-threatening illnesses such as cancer, caregivers are confronted with physical and emotional challenges [1,2]. Some studies report that the impact of a cancer diagnosis is greater on family members than it is on patients [3]. During the course of illness when cancer patients are not admitted to the hospital, family caregivers assume significant roles to support the patients. However, caregiving does not cease during hospitalization [3]. Caregiving becomes a full-time job once the patient needs assistance with even the most basic activities of daily living due to the effects of the disease, the treatments, or the combination of cancer and comorbidities.

Caregiving is a phenomenon that has increased in importance during the past decade. Providing care to cancer patients is demanding. Caring roles and responsibilities start when cancer is diagnosed. The complexity and uniqueness of the care giving to cancer patients varies depending on the type of cancer, stage of the illness, and type of cancer treatment. Care giving continues and can extend for several years until the cancer is cured or takes the life of the afflicted person. Supportive activities include household tasks, emotional support, and managing money [4]. Cancer patient care has both positive and negative impacts on the caregivers. Many caregivers experience a high level of satisfaction from their caring roles; conversely, many report a significant burden to their physical and psychological well-being, economic circumstances, and social and personal relationships [3].

Experience is subjective; it gives meaning to each individual’s perception of a particular phenomenon and how that individual consciously reacts to it [5]. The experience cannot be objectively measured by others; therefore, no measures can be used to examine individual’s experience. Although there have been a number of studies on caregivers of cancer patients, research studies that sought to quantify the effects of formal caregiving for the caregivers failed to successfully provide empirical understanding of the nature or essence of the caregiving experience. Quantitative studies cannot provide a description of the experiences of caregiving as clearly as studies that used a qualitative research design. A qualitative approach, such as phenomenology allows the researchers to examine, explore, and describe the lived experience of the persons who are caring for cancer patients [6]. Grounded theory is a qualitative approach that can also be used to explore and explain human experience [7]. Grounded theory approach has been used in many studies that were designed to explore the lived experience in dealing with a cancer diagnosis and treatment, as perceived by cancer patient’s family caregivers [8,9,10].

Over the last decade, a large number of studies regarding the caregivers’ experiences have been conducted and comprehensive models of cancer family caregiving were published. Some of those studies have been reviewed using different kinds of review methods, such as, literature review, systematic review, and critical review. Many qualitative reviews focused on exploring and describing a specific aspect of caregiving experience such as caregivers’ needs while providing care to people with cancer [11,12,13,14,15] and the impact of caregiving roles on the caregivers [16,17,18,19]. Among these reviews, some of the researchers showed their interest in a certain group of caregivers such as spouse caregivers [20,21] and formal caregivers—healthcare providers [22], while some of them focused on exploring a certain aspect of caregiving and coping strategies [23,24,25]. The reviews focused on the experience of caregivers of the patients undergoing some treatment [26,27].

Although scopes of existing reviews covered many aspects of the caregiving experience, the knowledge retrieved from the reviews has not yet presented a picture of the continuum of the experience. Thus, it has not been clear how the caregiving experience developed over time, as the caregivers were going through the course of a tragic illness like cancer. To explore this experience, researchers must look for the intention of the individuals’ actions and their perceptions and then described the phenomena that structured the experience [28,29]. The individuals’ intention is what they were trying to do in the situations they were facing. Because the experience is grounded in the life-world—the world as experienced by the individuals in their particular perspective [30], it is necessary to explore and describe how the individuals perceive the situation [29]. When caregiving is the phenomenon of interest, the phenomenon will be comprehensively understood only if the caregiving experience is well described. Thus, the cancer caregivers’ experiences are not apprehensible by a conceptual model although a conceptual model of cancer in a family caregiving experience has been developed and the relationships between and within the model elements are well described.

The aim of this present systematic review and meta-synthesis was to draw together the findings of qualitative research into a composition of the cancer caregivers’ experience. The findings of qualitative studies relevant to the caregivers’ experience were collected and synthesized carefully to gain a better understanding of the caregivers’ experience. Unlike other systematic reviews which were limited to the studies of caregiving at a certain course of cancer or in a certain group of caregivers, the present review—systematic review and meta-synthesis—put together the findings of cancer caregivers’ experience regardless of specific cancer or specific relation to the cancer patient, in order to get a comprehensive understanding of the caregiving experience. The researchers were interested in exploring the experience of the caregivers that began when they assumed their caregiving roles—their loved ones were diagnosed with cancer—and how they carried on these responsibilities throughout the duration of cancer treatment. The duration was from diagnosis until the treatments were completed or until the disease progressed and the purpose of the treatment had shifted from curative purposes to supportive purposes. The findings of this review show the components of cancer caregiving phenomena, the context of the caregiving experience, and the caregivers’ experience of caring for the patients with cancer.

The findings of a synthesis review provide more qualitatively rich evidence when compared to the original findings available from individual qualitative studies [27]. Knowledge generated from this systematic review will provide a comprehensive understanding of caregivers’ experience, including what the caregivers lived through, the nature of their everyday thoughts or attitudes, coping strategies, help-seeking behaviors, and concerns while giving care to cancer patients. This knowledge will help healthcare providers, especially nurses, respond in more meaningful ways when providing supportive services, or interventions to meet the needs of caregivers. When qualitative findings are rigorously and systematically reviewed, questions that pertain to individuals’ behaviors and perspectives will be more fully addressed [31,32]. The expanded findings can be used for guiding clinical practice and policy formation [33] Understanding the caregivers and identifying their specific needs is essential for healthcare professionals who are working with cancer patients and supporting the patient’s caregivers.

## 2. Research Questions

A collection of qualitative studies on caregivers’ experiences of caring for cancer patients, their perceptions, and responses to the situations in their daily lives has been conducted. This study focused on two specific review questions: (1) “What common themes pertaining to caregivers’ experience can be derived from the results of existing studies?” and (2) “What expanded knowledge can be gained about the caregivers’ experience from these common themes?”

## 3. Methods

### 3.1. Search Strategy

Studies were identified via electronic searches and reference lists from eligible studies. Four major databases, including CINAHL, MEDLINE, Academic Search, and Science Direct, were searched between October and November 2014. The researchers also used a Thai database of the Thai Library Integrated System (ThaiLIS). The ThaiLIS database allowed for searching full text of theses, research papers, articles, or other documents from all educational institutes in Thailand.

Keywords searched in titles and abstracts included: (1) experience; (2) cancer; (3) caregivers or carers or family members, or partners, or spouses; and (4) qualitative research or qualitative study, or descriptive research. The search was limited only to full text articles. Direct quotes from participants in the original studies were required for data analysis in order to preserve the meaning from the original text as interpreted by the authors or as raw data [34]. Additionally, the age range for searching was studies for participants between 19 and older.

### 3.2. Inclusion and Exclusion Criteria

The population eligible for inclusion was family caregivers, including parents, spouses, children, and next of kin, who were the main caregivers for cancer patients in their family. When patients and their caregivers were recruited, the studies were included only if the researchers presented data pertaining to caregivers’ experiences separate from patients’ experiences. Studies were excluded if the participants were interviewed after the death of the patients. Since the aim of this systematic review was to gain more understanding about the experiences of the caregivers while they were caring for cancer patients, it was necessary to exclude the bereaved caregivers who were no longer caring for cancer patients.

Studies were included if qualitative research methodologies were used. These methodologies included, but were not limited to, phenomenology, grounded theory, and ethnography. The included studies were also required to present data relating to caregiving experiences for a patient with cancer. Different qualitative methodologies can be combined for meta-synthesis if the studies aim to describe and explore the phenomena of interest [35]. Recently, different qualitative methods of a similar approach were included in meta-synthesis. The studies with the same methods were grouped together first for the initial examination before attempting any sort of synthesis between methods [36]. Although “qualitative research” was a keyword for searching, when the search results showed a mixed-design study, the study was included if qualitative and quantitative data were analyzed separately.

Studies were excluded if the patients were terminally ill and at the end-of-life stage, or if patients were receiving only palliative and supportive treatments at a terminal stage of the disease and no longer undergoing any active cancer treatments that aimed for cancer curation. Caring for patients who are in the final stages of life-limiting illness is a unique experience. Studies that focused solely on the symptom experiences, such as pain and cachexia, or the experience of receiving certain treatment procedures, such as nasogastric tube feeding, or attending certain interventions as parts of cancer treatments were not included because they were not focused on the experience of caring for cancer patients.

Moreover, studies published in English or Thai were included because the mother tongue of the researchers is Thai. The Thai researcher’s doctoral degree from an American university also ensures a high level of English proficiency. This bilingual competence helped to ensure that the translation would carry the meaning and the tone of the original text, while still remaining culturally sensitive. Discussion of the translations with a native English speaker fluent in both Thai and English was an additional means also used to ensure that translations conveyed substance and best meaning in both languages.

### 3.3. Search Results

**Figure 1:**
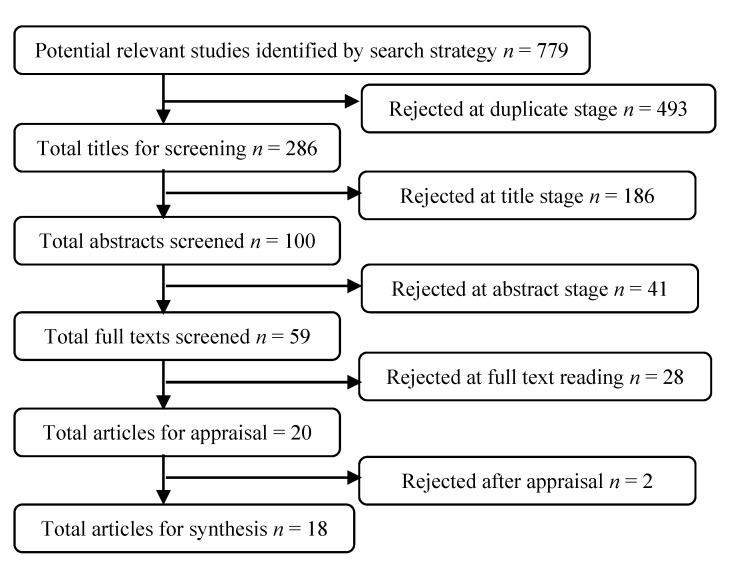
Flow diagram showing systematic review protocol.

Initially the search strategy yielded 779 articles. These articles focused on caregivers’ experiences of caring for cancer patients using qualitative research methods. After screening titles and abstracts, 59 articles remained for review. Their full texts were obtained and reviewed to determine whether the inclusion criteria had been met. The researchers read the articles and independently assessed them. A total of 28 articles were rejected for the following reasons: the patients were cancer survivors [37]; the patients were survivors with a recurrence [38]; the interviews were conducted after the death of the patients [39] or one year after the treatment was completed [40]. Studies were also excluded if the patients were terminally ill and receiving palliative care before the interviews [41,42,43,44,45,46,47,48]. Two studies were rejected because the experiences were not primarily about caregiving [49,50,51,52,53,54,55,56,57,58,59,60,61,62,63]. In one study, almost half of the caregivers were not actively providing care to the patients because the patients were at the point of remission or had passed away during the interview [64]. The remaining 20 articles were used for analysis (Figure 1).

### 3.4. Quality Appraisal

The quality of qualitative research was assessed mainly according to the Critical Appraisal Skill Program. (CASP) [65], an appraisal tool for qualitative research evaluation incorporating criteria adapted from an existing set of criteria proposed for evaluating qualitative research [66,67,68]. The criteria covered four areas: rigor, method, credibility, and relevance. Ten questions based on the CASP were used to identify good qualitative research assessment. Two screening questions were utilized to consider the aim and methodology of the studies. The other questions pertained to the details of the research methods to ensure the quality of the qualitative research including the research design, recruitment strategy and sampling, data collection methods, researchers’ role as a research instrument, ethical issues, rigor of data analysis, reporting of findings, and value of the research.

### 3.5. Data Extraction

Data extraction is a straightforward component of a systematic review. The focus and range of data extraction depends upon the purpose of the review. However, key terms for the extraction are based on the participants, interventions, comparisons, and outcomes of interest, depending on the purpose of the review [69]. Therefore, the first and second researchers read the studies independently in order to find key concepts from the studies. The findings in relation to caregivers’ experiences pertaining to caring for cancer patients were coded into first-order and second-order constructs. The first-order construct consisted of direct quotes from participants of the original studies based on their own experience; whereas, the second-order constructs were the original authors’ interpretations of the participants’ accounts [27]. When data were extracted, it is important that the meaning from the original text was preserved [56]. To preserve the meaning from the original studies, the researchers tried to code the second-order construct by using the participants’ words or the original authors’ own language.

When the extraction process was completed, the researchers read and reread the second-order constructs and then developed a theme to describe the caregivers’ experience. The themes were developed based on the researchers’ interpretations of the participants’ experience presenting the second-order constructs. At this stage, the researchers set aside preconceptions derived from previous knowledge regarding the caregivers’ experiences, particularly caregiver coping experiences. Setting aside or bracketing is a method used in phenomenological study to ensure that the data gathered from the participants and the experiences were derived only from the participants [29,30]. Setting aside preconceptions prevented the researchers from using the constructs or components from existing conceptual models when setting up the theme categories and writing the narrative summaries of each category. Thus, although caregivers’ experiences such as burdens and stages of coping had already been described in the literature, they were not used in the data synthesis stage. However, it is possible that the words or phrases derived from the original studies were similar to the terms used in existing conceptual models. The information set aside was then reintegrated into a discussion of the experience [29].

In order to establish the validity of the data extraction, completed data extraction forms from the two researchers were then compared. If the assessment results of some studies were different, the researchers worked together to decide whether to include or exclude the study. However, there was no major difference in data extraction found.

## 4. Findings

After the appraisal process, two articles were rejected. One was rejected because the interviews were not audio-recorded [70] and another was rejected because, although the interviews were recorded, participants’ quotes were not provided [71]. Eighteen eligible articles remained for data analysis. To create a meaningful and valid meta-synthesis, it is recommended that at least 10-12 studies should be purposely included in the synthesis [72]. Therefore, 18 articles was an appropriate number for this systematic review.

The individual studies that were included in data synthesis described the caregiver’s perceptions of caregiving, needs, and the impact caregiving had on them. These studies still left some gaps in understanding the caregiver’s experience. To enhance understanding of the caregiver’s experience, the researchers used a technique of phenomenological data analysis to analyze the data pertaining to the caregivers’ experience. The context surrounding the experiences and the phenomena that structured each experience were explored and described. Because the purpose of this analysis of qualitative research was to synthesize the findings into a thickly descriptive and comprehensive product [73], so the aim of this review was not to discuss every detail of the findings from original studies; only the portions of the results that related to the aim were included.

The experiences of caregivers who were caring for cancer patients described in this section consisted of two components: the context of the experiences and the phenomena that structured the experience. As data were extracted, the researchers looked for the intentions of caregivers—what they were trying to do while they were caring for the cancer patients. Intention consisted of conscious acts or intentional acts [74]. These acts were the result of individuals’ interpretations of present events and expectation of future goals [28,75]. For example, one intention found was trying to balance emotions. This intention consisted of intentional actions including staying positive, searching for hope, getting their mind off a straining situation, and comparing their situation to the worst case of others’. Intention was used to describe the caregiving phenomena.

Actions are structures of experience that link perceptions and intentions [76]. When participants described an important activity they were doing, they often went on to explain why it was important; thereby revealing data relevant to the personal-social context of the experience [30]. Personal context is a specific context layer that has a direct influence on an individual’s actions, whereas a broad context layer has an indirect influence on an individual’s behaviors [29]. A broad context includes a social construct such as social background such as belief, religious, social support, and healthcare systems [29]. Therefore, as the researchers were looking for the caregivers’ intentions, the researchers were also trying to find an explanation of why the caregivers performed certain actions. For example, the researchers found that the caregivers were trying to balance their emotions because they were having a hard time dealing with emotional devastation. Thus, “having a hard time dealing with emotional devastation” was the context of the phenomenon “balancing my emotion” and it was a specific context that directly influenced the caregivers’ actions or behaviors.

The common themes pertaining to caregivers’ experience derived from the results of existing studies were categorized into the three sets of contexts and phenomena relevant to the experience of the caregivers who were caring for cancer patients were extracted from the original studies (Table 1).

**Table 1:** The Context of the Experience and the Caregiving Phenomena.

Context of the Experience	Caregiving Phenomena
**Set One**	**Set One**
1. Having a hard time dealing with emotional devastation	1. Balancing my emotion
1.1 Being in shock	1.1 Staying positive
1.2 Being in denial	1.2 Searching for hope
1.3 Being in panic	1.3 Getting my mind off it
1.4 Being fearful	1.4 Avoiding the discussion related to cancer
**Set Two**	**Set Two**
2. Knowing that a caregiving job was laborious	2. Keeping life as normal as possible
2.1 Feeling helpless	2.1 Living one day at a time
2.2 Feeling overwhelmed	2.2 Rebalancing life at every step
2.3 Feeling inadequate	2.3 Getting out of the situation temporarily
2.4 Feeing uncertain about the future	
**Set Three**	**Set Three**
3. Knowing that I was not alone	3. Lifting life above the illness
3.1 Having a closer relationship	3.1 Maintaining a meaningful life
3.2 Having support from family and friends	3.2 Accepting support from family and friends
3.3 Having God watching me	3.3 Leaving it in God’s hands

### 4.1. Context of the Experience

The contexts of the experience described the caregivers’ negative and positive feelings about cancer and caregiving. Caregivers’ expressions showed that being a caregiver was very burdensome—requiring tremendous physiological and psychological effort. Although most of the studies showed the negative aspects of caregiving; several studies discussed the positive aspects of the caregiving.

#### 4.1.1. Having a Hard Time Dealing with Emotional Devastation

“Emotional devastation” was used to describe the emotional impact of the cancer, particularly at the initial diagnosis [77]. The emotional impact of cancer was the most intense during the initial cancer diagnosis. Caregivers described it as their world being “turned upside down” [9] and “being slapped in the face” [10,77]. A husband of a woman with ovarian cancer recalled the first 24 h as being the most difficult. He said, “We came to the hospital and [they] told us that she did have cancer and it was probably not early cancer, and we were both so devastated and we just cried together for the first hour.” [77].

Although the intensity of the feelings declined over time, but stayed with the caregivers throughout the treatment until the end of life of the patient. The feeling of devastation could be exacerbated when the caregivers and patients had not yet discussed the extent of the disease, progression, and treatment with their physician [77,78]. Most of the caregivers described their feeling when they initially learned about the cancer diagnosis as “being shocked” [77,79,80] and “feeling terrified” [80] due to the thoughts that accompanied the news. At this stage, they were in denial and feeling panic, fear, and sadness.

##### Being in Shock

Receiving the news that their loved one was diagnosed with cancer was a shock because the caregivers perceived that cancer was a life-threatening disease. In many cases, the caregivers thought if the disease had been diagnosed earlier, it might not have progressed or may have been more treatable [2]. A wife of a prostate cancer patient said, “*The doctor kept saying it was muscular; he [patient] must have strained himself till it got too late. It [the cancer] got a lot worse than it should’ve been*, *it should have been much more treatable…*” [2].

##### Being in Denial

The diagnosis was unexpected, particularly in the patients who had no family history of cancer, having no signs, or any risk behaviors that caused cancer. Soon after being shocked by hearing the bad news, caregivers were in a stage of disbelief and in denial. It was hard for them to accept that this was actually happening to them. A daughter of a breast cancer patient said, “*Well, at first, I was in denial. I just didn’t want to—a lot of things were going through my mind… it took me a while to really come to terms with reality, that in reality it was cancer*” [79]. Along the course of the treatment, the feeling of being in denial continued [8] until they accepted the fact that the patients had cancer. At this stage, the caregivers avoid discussing sensitive issues such as treatment options, which increased emotional distress to both the caregivers and the patients [9] and delayed the treatment.

##### Being in Panic

This feeling was so intense that it temporarily immobilized the caregiver from taking any purposeful action. As one daughter of a breast cancer patient said, “*[Y]ou’re panicky; you’re splattered—your head is splattered all over; your thoughts are jumping—and you don’t know what direction to go into, you’re just wild.*”[79]. The caregivers felt panic because they were not certain about the patients’ well-being and the cancer treatment effects.

##### Being Fearful

Awareness of the inevitable death of cancer patients awoke worries about what their own future life would be like. Although this feeling diminished gradually, for some caregivers these fears and worries endured [79]. Similar to panic, fear diminished the caregivers’ ability to concentrate and process information [81]. There were two types of fear: fear of losing the loved one and fear of the loved one’s survival.

*(1) Fear of Losing the Loved One.* Hearing the diagnosis of cancer resulted in strong feelings of fear. Some caregivers perceived a cancer diagnosis as an impending death [2] and they could not live their lives as they did before the cancer diagnosis without acknowledging the fear of death of their loved one [82]. Because having lived a long life together meant a long mutual history that connected the mates together; therefore, it was hard for the caregivers to think about losing their partner and facing a future life alone—the loss was unbearable [83]. “*Without him there is nothing, no meaning. I mean, you’ve built up a life up together, you’ve made plans, there is so much to do ... so much left to do…*” [2].

*(2) Fear for the Loved One’s Survival.* Many studies reported that the interviews were emotionally laden and the caregivers were tearful as they described their fears for the patient. Unlike the fear of losing a loved one, this type of fear was due to the caregivers’ concerns for the patients’ health and well-being. A daughter of a mother with breast cancer said, “*…if someone would tell me she will be a hundred percent fine, which I don’t know if they ever will, I’m going to be scared for her life…for her health and I’ll be scared of something happening*” [79].

#### 4.1.2. Knowing that Caregiving Jobs were Laborious

Caregiving tasks and responsibilities, which included providing physical support and psychological support for the cancer patients, were demanding. Physical support included helping the patients with their daily activities, managing disease symptoms, and treatment effects. Findings from the original studies showed that while dealing with physical needs of the patients, the caregivers had also need to deal with the patients’ emotion. The caregivers expressed how they felt with the caregiving job as follows.

##### Feeling Helpless

The caregivers felt helpless when they could not keep the patients from suffering or they did not know how to take care of the patient [78]. Some caregivers expressed the feeling of helplessness when witnessing the patient’s deterioration or seeing the patient suffering from side effects of the treatment but being unable to help alleviate them [10]. Being unable to understand medical terminology and not being included in the process of treatment also caused the caregivers to feel helplessness because they could not provide adequate care or support for the patients [84]. One caregiver explained, “*We needed an interpreter when he was diagnosed, all of this was a foreign language, and we were not hearing anything. That was stressful, when family asked me questions; I didn’t know what to tell them, because I didn’t know what was said…*” [80]. Some caregivers expressed that their needs were not recognized and they did not received adequate support to help them overcome the cancer situation. A wife of a man with prostate cancer said, “*He was wasting away in front of me, and I just really wasn’t clear what I was supposed to do and I was ringing the surgery and saying ‘you know I think somebody needs to see him’ and all week the doctor didn’t come*” [85].

##### Feeling Overwhelmed (by the Caregiving Job)

Becoming a caregiver was a second full-time career as caregiving had become the priority in their life [77]. By three months, the caregivers were physically and emotionally exhausted [78]. The caregivers reported that their living routines changed and their lives were more restricted [9].

Living routine changes were caused by increased work and responsibilities when the caregivers became an advocate for their patients and took over tasks that used to be done by the patients inside and outside of the home. Most of the tasks inside the house were household chores and work to help the patients met their daily living needs. The normal household duties at home were shifted to the caregivers, particularly spouses; as a result, the caregivers became extremely busy and found it difficult to continue with their daily lives [86]. Tasks outside the house included being note-takers at oncology visits, navigators of the insurance system, medication distributors, appointment schedulers, and a spokesperson to family and friends regarding their spouse’s health [80,85].

The responsibilities increased and the caregivers were limited in their activities when the patients declined in their ability to take care of themselves. The patients were more dependent as the cancer progressed and the treatments started to have more effects. When the treatment started, it brought the caregivers’ and the patients’ normal living to a standstill [9]. Their plans for the immediate future had to be changed in order to make a new plan to match cancer treatment schedules. The less physically mobile the patients became, the more the caregivers were limited in their activities [10]. The caregivers explained how their life had been pushed to the side and became limited, as they focused all their attention on caring for the patient and put their lives in the background [80,82]. A female caregiver said, “*To hell with myself, I will do anything for him, even if that means putting myself last*” [80]. Trying to meet the patient’s needs and placing their own needs last eventually caused caregivers to feel overwhelmed and burned out.

Studies showed that the caregivers devoted themselves completely to the task of caregiving. One man said, “*I did everything from meals to wheels…*” [80]. Their daily lives and plans were disrupted and often set aside [81]. They could not participate in other activities because cancer patients needed a lot of assistance. Consequently, the caregiving job affected the caregiver’s daily routine [10]. They may not get enough rest or have time for any leisure activities and neglected their own needs and their own health which consequently caused them to become ill [87,88].

As the caregivers went through a pile up of compounding hardship, they described that it seemed as they could not “*catch a break*” [80]. However, they were reluctant to take a break because it made them feel guilty, as one of the spouse caregivers said, “*I felt guilty for wanting to take a break, if I was not there for him, who was going to be?*” [80] As a consequence, they missed participating in social activities because they had to be with the patient all the time at home and at the hospital. Over time they were also exhausted, overwhelmed, and felt as if they lived in a state of suspension.

The caregivers also felt overwhelmed with the patients’ hospitalization and treatment options, which called for difficult decisions to be made. When there were multiple complications in treatment and multiple hospitalizations, it was difficult to process the information given to them. They felt that it was difficult to sort out choices when the diagnosis was initially made and there were various treatment options. One couple described the experience: “*If you go to a surgeon, they want to cut you open, and if you go to an oncologist, they will want chemo... so everybody’s got their own approach. As we said earlier, we wanted to have some closure on it. Just tell me what to do, but they don’t say.*” [81].

##### Feeling Inadequate

Many caregivers felt that they had never done enough to satisfy the patients or meet their needs because the patients’ demands increased and the information about cancer and it treatments they received from healthcare providers were not adequate. After becoming a caregiver, the caregivers experienced a transformation of their roles and had to adopt a number of new caregiving activities. In addition to household responsibilities, caregiving tasks in the house included preparing meals, changing cloth, making beds, helping the patient get to the toilet, moving the patient from one place to another. Although the caregivers had done a lot for the patients, they still felt that their support was not enough and had not yet met the needs of the patients and they questioned their capacity to provide sufficient care [82].

The caregivers felt that the support from healthcare professionals was not sufficient [77]. The caregivers acknowledged themselves as important links between the patients and the professionals, especially when the former was too ill to communicate with them. They were all involved in the contact with the healthcare system [83]. However, some of them described themselves as being treated like strangers and as not having been treated as a significant person. Healthcare personnel were “*professional*”, but distant [81]. They often felt inhibited when they came into contact with healthcare professionals [85]; “*nobody cares for the caregiver*” [80]. One caregiver said “*I felt like a second-rate citizen*” [80]. Without knowledge and training, the caregivers felt that they could not provide adequate and appropriate care to the patients [78,88]. The caregivers’ needs included information and emotional support. They found that support from health professionals was inadequate [83].

##### Feeling Uncertain about the Future

The caregivers’ everyday life was filled with the feeling of uncertainty about the future. They felt uncertain about what was expected when the patients’ conditions were unpredictable. One of the caregivers described the uncertainty of the situation as “*without symptoms one day–just the opposite the next day*” [89].

This feeling occurred beginning when they were waiting for the confirmation of a diagnosis and continuing throughout the course of the patients’ lives. Lack of understanding about treatments made it difficult to decide the best options for the patients. The caregivers felt that if they knew the effects of treatments or understood the process of treatments, they should have been able to provide physical and mental support to the patients [84]. Moreover, they found that it was difficult to deal with the patient’s behavior which became unpredictable during the course of the illness. Some of the patients became irritable and angry with caregivers [9]. As a consequence, the caregivers found this very distressing, as they did not know how to handle the situation. The caregivers also described a sense of insecurity regarding the future and being unable to make long-term plans as they were waiting month-by-month for the results of the next examination. They could not determine whether the result of the next examination would be good or bad. However, many caregivers expressed a sense of urgency even when the results of testing were good [81]. However, they felt more certain about the future when the treatment ended as one of the caregivers said, “*It has been a very long journey… yet… I see the future as bright… very bright. Everything will get better.*” [9].

#### 4.1.3. Knowing that I was Not Alone

Although the studies showed that the caregivers had negative attitudes toward cancer caregiving, positive attitudes were also found. A set of the phenomenon “knowing that I was not alone” showed a positive perspective on being caregivers of cancer patients. Compared to the thoughts at the beginning of the treatment, the caregivers’ thoughts were more positive when the treatment ended [9]. Although the caregivers’ lives had been changed drastically and the emotional impact was very intense, the caregivers still acknowledge positive aspects of life. The caregivers’ expression “seeing good in a bad situation” represented a positive attitude toward the situation. When the caregivers focused more on the positive aspects, they realized the unseen benefits of the disease. Some of the benefits the caregivers noted were: (1) having a closer relationship with their relatives or spouses; (2) having support from family and friends; and (3) having God watching me.

##### Having a Closer Relationship

As husband and wife, spouses, and next of kin realized the closeness in their relationship. A cancer diagnosis reinforced the strength of support in the family and the caregivers realized that their marriage had changed for the better. For faithful mates, it is important to be seen as a unit during the disease process [83]. One spouse said, “*We actually do talk to each other, we are closer now than we have ever been*” [80]. A husband of a cancer patient said “*It’s brought us a lot closer…we’ve both gotta deal with this, not just her, not just me, we both do… we have made more efforts to spend more time together…we have made a point of doing more things together*” [77]. They realized that simply spending time together was helpful, not only to those who were sick, but also to themselves. By spending time together, the caregivers felt that they became closer to their sick loved ones and sometimes they discovered new, previously-unnoticed characteristics about the relatives that eased stressful situations. Developing this kind of relationship brought meaning to the caregivers’ life [87,88,89]. They acknowledged an intensified family relationship. One daughter said, “*It just made me more aware that I need to love every minute I have with her*” [79].

##### Having Support from Family and Friends

The caregivers’ daily routine was disrupted by travelling to and from the hospital and doing household tasks. Family and friends were described as a significant support because the family and friends not only shared the caregivers’ burden, but they also helped to support cancer patients physically and emotionally [88]. Family members could alternately be with the patients and assist with household responsibilities. With this support, the caregivers could resume their normal activities, such as returning to work and taking care of the children. Support from family and friends had been helpful, the caregivers thought that burden would have been harder without this kind of support. A husband of a woman with ovarian cancer described: “*I’ve realized how caring people are…I think it’s just the realization that this is nothing that [we] are going through by ourselves*” [77]. Similarly, a spouse of an oral cancer patient said, “*The relatives… do all the practical work…even something as simple as washing dishes, cleaning house, doing the wash, grocery shopping… that takes a lot of energy*” [9].

##### Having God Watching Me

For some caregivers religion was a foundation of psychological and spiritual strength. They had faith in their religious beliefs. One caregiver said, “*God watches out for me…*” [90]. Some caregivers explained that they gained strength from their religious communities and prayer groups. They found support in the sentiments offered by fellow members at their places of worship [81]. They believed that the family, friends, church, and community support they had were “a God send” [80]. God watching was not only applied to a present life, but it was also applied to an afterlife. It was a great comfort for them when people said to them “*I’ll pray for you*” [81]. A religious caregiver expressed his belief, “*I believe in an afterlife… I think that’s one of the reasons it doesn’t worry me a lot… (not) bothering me, because we’ll have a better life then*” [9].

### 4.2. Caregiving Phenomena

Three phenomena of the experience were found. The phenomena showed the caregiver intentions—what they were trying to do when they were dealing with the situation as caregivers to cancer patients. While the context of the experience showed the caregivers’ perceptions of the situation, the phenomena showed how the caregivers dealt with caregiving situations.

#### 4.2.1. Balancing My Emotion

Living in close relation to a serious illness like cancer was physically, mentally, and emotionally draining. The caregivers described that they needed to consciously balance the positive and negative aspects of their lives. Balancing emotions was different between the caregivers who accepted the situation compared to those who avoided reality. Those who accepted the situation tended to adjust to cancer and caregiving consequences; therefore, they were not burned out during the caregiving process. In contrast, those who avoided the fact often guarded themselves from cancer-related situations.

##### Staying Positive

One caregiver suggested that “*you have to prevent negative thoughts from creeping into your life…*” [90]. Feelings such as being in shock and disbelief lasted only a short time; whereas, other feelings, such as panic and fear were more long-lasting [79]. One caregiver said, “*You have to try and be positive, and not just talk about the worst that can happen, not succeeding, not getting better. Because then you end up thinking negatively. Even if… many times… you may think... how awful… what if it doesn’t succeed. So you have to think positively* [9]. Another caregiver stated, “*I feel fortunate, others have it way worse than we do*”. “*We really are lucky if you look at the big picture,*” and “*we have had so many good years.*” [80]. Instead of dwelling in sadness and sorrow for their life and the patients’, some caregivers compared their situation to other people’s who had more problems. One caregiver said, “*There are people with more serious problems. We have the advantage of no financial problems, I’m healthy and I can go to the hospital by car*” [10]. The caregivers not only tried to be positive themselves, but when the patients were not able to be positive; the caregivers tried to find a positive story and proposed their thoughts to the patients.

##### Searching for Hope

Hope was tangible and important to the daily life of the caregivers. It gave the caregivers courage to support their loved one [90]. “*Hope is about making the best of a bad situation and moving on*” [90]*.* One caregiver said, “*Engage hope... as [hope]... is working for you, so that you are imagining what can be done and then doing it*” [82]. Hope was not only important for the caregivers to have a positive attitude, but the caregivers believed that it was also helpful for the patients.

Religious beliefs were important regarding hope, particularly when the chances that the cancer would be cured were minimal [9]. Having faith helped the caregivers balance their emotions. A male spouse of a breast cancer patient said, “*it does give me hope that there are—well, that you know, God watches out for me and for my wife and for my kids*” [82]. Another caregiver said, “*…I know there’s no cure, unfortunately,* *but they can prolong your life if the treatment works. And, as I say, if it’s only another five years… and perhaps doing things we’ve never done… So I’m hoping that, with the help of God...*” [9].

##### Getting My Mind off It

The caregivers found that when they felt overwhelmed, frustrated, and exhausted from caregiving roles and cancer effects, getting their thoughts off an unpleasant situation helped them to balance their emotions and recover their strength. When their thoughts were still and things were quiet, their anxiety was the highest [80]; therefore, they had to stay busy [88]. The caregivers who accepted the situation used activities such as journaling, therapy, prayer, self-reflection, hobbies, music, and exercise [80,82] in order to recover their physical and psychological strength. The caregivers found that these activities distracted them from an unpleasant situation and helped them to recover their strength. One caregiver said, “*Get up and get something done, it takes you mind off it, that’s my cure for everything, if there’s something on your mind don’t sit, get up and do*” [21].

##### Avoiding the Discussion Related to Cancer

The caregivers who had not yet accepted the situations tended to avoid the fact and often guarded themselves from cancer-related situations. They avoided discussions about cancer because cancer-related topics were heartbreaking. Talking about it could even upset them as much as it did to the patient [10,80]. Some caregivers found that emotional distress was exacerbated when the caregivers and the patients discussed the extent of disease and prognosis [78]. Thus, it “was an unspoken rule not to mention the cancer” [8]. Most of the caregivers admitted to not bringing up emotions related to cancer when talking to the patient because they were afraid that it might upset the patient [80]. They said “*we did not discuss the ‘C’ word*” [80], and they were unwilling to discuss issues such as treatment decisions, financial factors, wills, death, and funeral arrangements, [79]. One caregiver said, “*Since he has been diagnosed we have never said the “C word in conversation…I don’t want to think about the outcomes, and he did not seem to want to discuss them*” [80].

#### 4.2.2. Keeping Life as Normal as Possible

“*Normalization of their own care-giving*” was a typical way the caregivers lived their lives [10]. After three month of caregiving, some caregivers were trying to get back to a sense of normality. They tried to “*keep on living as usual*” and “*take the days as they come*” [9]. They were aware that their life together with the patient was very short and they were not certain about the limited period of time. Hence, it was important that life continued as normally as possible, despite the major event resulting from the effects of cancer [83]. One caregiver said, “*As a family you just find out what you have to do and you just stick to it, all the while you search for normalcy*” [80]. Although they were aware that their life would have never been as normal as it was, they tried to resume a normal life for themselves and the patient.

##### Living One Day at a Time

They lived their life “*one day at a time*” [80], as a caregiver of an oral cancer patient stated, “*I guess we’ll just keep on living as usual. Maybe take the days as they come a bit more*” [9]. There were caregivers who no longer focused on a cure, which was a long-term goal, but on stabilization, which was a short-term goal, [10,86] showing that they were living one day at a time. This aspect did not only cheer up the caregivers, but the caregivers also used it to support the patient, as well. A male spouse said, while he was driving home after a fourth chemotherapy, “*We have been here four times. She does not have to get the fifth next week. Now we have been here once again. So maybe, if we go through it another three times, we might not have to come once again. So I always find good prospects*” [10].

##### Rebalancing Life

Rebalancing life was important because many times the needs’ of patients was the priority and the caregivers put themselves last. As a consequence, they forgot to take care of themselves. To find balance, the caregiver described that importance of looking after themselves. One caregiver said, “*It’s utterly important to take care of myself and make sure that I’m healthy, so that I can take care of him (my father)*” [87]. When the caregivers felt overwhelmed by the caregiving job and felt physiological and psychological exhausted, they realized that they needed a break—time to do something for themselves—and they had to make it happen. One caregiver said, “*You’ve got to make it happen yourself…other people aren’t going to look after you; you look after your own self and get on with it*” [90]. Some caregivers reported making positive changes to their health; therefore, they had strength to take care of cancer patients. The changes included quitting smoking and eating more healthily. They realized that self-care was just as important as the care of the patients [80,82].

##### Getting out of the Situation Temporarily

Some caregivers stated that being away from the patient for a short period of time helped them to recover from physical and emotional exhaustion [9,88,89]. One caregiver said, “*When (my wife) got sick…I would just always worry about her. And I would try to do things to release stress or blow it off or whatever…*” [94]. One way the caregivers chose that helped them to recover and gained back their strength was finding time and space for themselves to relax from their daily routine—always doing something for the patients. One caregiver said that it was important “*to get half an hour or an hour where you can go outside…then you have something to give to the sick when you are back home because you are there 24 hours*” [89].

#### 4.2.3. Lifting Life above the Illness

This phenomenon is the experience that is one level beyond the others. “*Lifting life above the illness*” was an optimal goal of the caregivers. It referred to the ability of the caregivers to overcome the burden of cancer; therefore, their life was no longer altered by the cancer and its treatments [80]. Only caregivers and patients who had a strong relationship and religious beliefs were able to achieve this stage.

##### Maintaining a Meaningful Life

“*Not taking life for granted*” [8] is best described as a component phenomenon of “*maintaining a meaningful life*”. “*Maintaining a meaningful life*” was identified as important; however, the caregivers found that to fulfill their caregiving role, it was difficult to maintain their everyday lives [89]. Over time, the caregiver became stronger and they were able to develop strategies to deal with the difficult situation they encountered. The caregivers who accepted the situation were able to maintain a meaningful life and being conscious of value of life [89]. After they were able to maintain a normal life for themselves and the patient, they re-evaluated what was important to them and set a goal for their life [80,87]. When they found the meaning, they lived their life accordingly. As one caregiver said, “*Cancer has forced me to re-examine my own life*” [80].

The caregivers found the value of their life and their time together with the patients. Particularly for spouses, life was more meaningful when they were living with their loved one. They experienced the feeling of “love” they had for the patient; and therefore, they wanted to do extra beyond the routine caregiving duties to express their feeling of love verbally or non-verbally. A husband of a woman with ovarian cancer expressed: “*I think it’s helpful for me [to go with her to chemotherapy treatments] just because I see there is something being done for the person I love*” [8].

A meaningful life could be “*spending quality time together*” [81] or “*doing things together*” [9]. The caregivers wanted to support their loved one as much as they could. However, not every caregiver achieved their goal. Some caregivers stated that the patient did not want them to be involved in the treatment process and did not explain enough information for them to understand the situation. For example, a husband of a woman with breast cancer said that his wife wanted to stay with her mother and left him and the children at their house and he was not happy with his wife’s decision [84]. When caregivers and patients went through illness and the cancer treatment together, they developed a “*strong alliance*” called “*together-relationship*” [10]. They understood each other and their relationship were strengthened. Caregivers who developed this kind of relationship found a balance between a role in supporting the patient, as well as maintaining themselves to the point that they did not struggle daily with the consequence of the disease. In contrast, for the caregivers who struggled, their life was disrupted by caregiving duties. As a daughter of a breast cancer patient said, “*I dropped my life…if I was working or had my own family my mum wouldn’t be here today…*” [78].

Caregivers who could lift their life above the cancer also had a sense of accomplishment, which was a perception of personal satisfaction [91]. They developed a sense of accomplishment when they overcame a number of challenges caused by cancer and treatment effects. Challenges could be: seeing themselves helping the patient feels more comfortable, being respected, being appreciated by their care-receiver [91], or being able to accept the consequences of cancer and treatments. One study reported that husbands of mastectomy patients perceived a sense of accomplishment when they found themselves being able to adapt to sexual disruption, to the possibility of the wife’s foreshortened lifespan, and to unanticipated changes in partner behavior, disposition, or interests [82].

##### Finding and Accepting Support from Family and Friends

Friends and family were described as important [83]. The caregivers described the value of having family and friends and accepting help from others [89]. Although the caregiver described the value of having family and friends and accepting help from others, some of them felt that their family was their responsibility and no one should have to look after their family [8]. When the caregivers were overwhelmed by negative feelings, they sought emotional and physiological support. They looked for someone to listen to their fears and needs [87]. For some caregivers, it was important to handle the situation themselves, so they were reluctant to accept help and support [83]. One caregiver said, “*The mums (mothers) at school…offered to help and scrub the toilet and things like that, but I felt really uncomfortable about people coming in... you feel you should be able to look after your family*” [8]*.* On the contrary, a caregiver who accepted help said, “*My girls made sure everything was spotlessly clean, meals cooked and everything else, and looked after us very well*” [8].

##### Leaving Life in God’s Hand

The caregivers’ religious beliefs demonstrated their faith in their God—Jesus, Buddha, and Allah. When the cancer caregivers felt hopeless and felt that they had nothing to hold on to because everything was very unpredictable, they prayed to their God. Praying was one way to strengthen their courage. One participant said, “*When I feel very stressed, I pray. It’s very helpful. It helps to reduce my worry”* [96].

### 4.3. A Relationship between Contexts and Caregiving Phenomena

Using a descriptive phenomenological data analysis method, the researchers tried to capture the overall essence of the experience. In addition to looking carefully for the caregivers’ intentions and the rationale that the caregivers gave for their intentions, the researchers put an effort into finding the link between the contexts and the phenomena that structured the experience. The contexts represented the caregivers lived world or how they viewed the situation while they were caring for cancer patients. Each context consisted of relevant contextual features; however, the contextual features were not necessary to have a linear relationship. Each phenomenon consisted of a set of its component showing the caregivers actions relevant to their intention. Table 1 showed three sets of the context and its relevant phenomenon.

#### 4.3.1. Relationship between Set One Context and Phenomenon: Having a Hard Time Dealing with Emotional Devastation and Balancing My Emotion

The context—“having a hard time dealing with emotional devastation”—consisted of four contextual features: being in shock, being denial, being in panic, being fearful. These contextual features showed the negative perception toward cancer and treatment. These feelings were dynamic and changing. The caregivers perceived that cancer was a life-threatening disease; thus, shock and denial. These feelings usually came with the initial diagnosis. Many studies reported that the intensity of the feelings declined over time, but stayed with the caregivers throughout the course of the treatment until the end of life of the patients. At stages of the disease and the treatment, the caregiver also felt panic and they were fearful. Some studies showed that although it had been two years since the cancer was diagnosed, the feelings of panic and fear had been sustained. This set of perception consisted of negative feelings. These feelings were not necessary to have a linear relationship and some feelings could exceed the others depending on the people and the situations.

The phenomenon—“balancing my emotion”—consisted of four component phenomena: staying positive, searching for hope, getting my mind off it, and avoiding discussions related to cancer. As the caregivers were having a hard time dealing with emotional devastation, they tried to balance their emotion. Dealing with the psychological distress, the caregivers tried to balance their emotion by thinking positively, searching for hope, getting their mind off of the situation. To make the best of the bad situation, the caregivers emphasize the value of a positive attitude [61] and the need to balance the positive and negative perspective of their lives [90]. They found the importance of having a positive attitude which helped them and the patients to overcome any difficulties in their lives. Moreover, they searched for hope. The diagnosis of cancer required the caregivers to be more aware of their hope and to engage or activate it to help them deal with their situation [90]. The positive attitude of the caregivers influenced hope of the patients. Hope sometimes related to religious beliefs. Having faith in the God they worship gave them hope and helped to balance their negative attitude and positive attitude.

Avoiding and finding distraction were other strategies to balance the caregiver’s emotion. They tried to get their mind off of the situation that caused them to feel distressed. When they felt that the negative feeling was unbearable, the caregivers tried to find distractions, such as housework, hobbies, and exercise that could disrupt their thoughts. The caregivers also prevented themselves from dwelling in negative perceptions about the cancer by avoiding communication about the topic related to cancer. Cancer-related topics caused emotional distress to both the caregivers and the patients. The caregivers who felt strained to talk about cancer were the group that had not yet been accepted the situation [80]. Although many of the caregivers used avoidance and distraction to balance their emotion, they were aware these strategies helped them temporarily.

#### 4.3.2. Relationship between Set Two Context and Phenomenon: Knowing That a Caregiving Job Was Laborious and Keeping Life as Normal as Possible

The context—“knowing that a caregiving job was laborious”—consisted of four contextual features: feeling helpless, feeling overwhelmed, feeling inadequate, and feeling uncertain about the future. These feeling were not separated; they could occur at the same time as well as one could be the cause of the others. This context represented another set of negative perspective on caregiving experience. These negative feelings occurred anytime while the caregivers were caring for the cancer patient.

Feeling helpless occurred either when the caregivers could not provide help or find help that was adequate and appropriate for the patients. Because of the nature of caregiving as a full-time job and that the patient was a center and the priority of care, the caregivers often felt that their support had not been enough for the patients. They felt overwhelmed, meanwhile; they felt helpless. Feeling helpless also occurred when the caregivers could not find a source that could provide them with appropriate and adequate help, particularly professional healthcare sources.

Feeling inadequate always related to the limitation of health information and healthcare services available to access. Health information was important, the caregivers thought if they could understand the cancer and the treatment, they would have known what to expect and how to deal with it. However, they felt that it was hard to get sufficient information. Some caregivers expressed the frustration caused by being unable to understand healthcare vocabulary. If they knew what to expect at each stage of the cancer and treatment, they would be more certain about the future.

Feeling uncertain about the future made it difficult for the caregivers to plan their lives. They were uncertain about the conditions of the patients; consequently, the caregivers could not make a plan for their long coming future. The caregivers emphasized that if they could be aware of unpredictable symptoms and understood the treatment effects, they could have managed the situation better and their lives would have been less distressed.

The phenomenon—“keeping life as normal as possible”—consisted of three component phenomena: living one day at the time, rebalancing life at every step, and getting out of the situation temporarily. This phenomenon was more physical focus compared to a phenomena “balancing emotion” that was a more psychologically focused phenomenon. This phenomenon showed how the caregivers responded to their perception which was dealing with a demanding caregiving job. 

Because of the unpredictable future regarding the patients’ conditions and the consequences due to the side effects of cancer treatment, the caregivers admitted that it was easier to make day-by-day living and plan. They realized that their future was uncertain; they could not make a long-term plan, but lived day-by-day. Meanwhile, they had to rebalance their lives; therefore; they did not dwell on the 24-hour caregiving job and felt overwhelmed. The caregiving job was not tidy and never ending. Most of a long-lasting job of caregiving involved household work and assisting the patients in the house; therefore, being in the house was stressful. The caregivers found that if they did not get out of the house, they would have forgotten about themselves including their needs and their well-being. When being in the house turned to an unpleasant situation for them the caregivers got out of the house and found something to do such as going to a store or visiting relatives in order to restore their physical and psychological strength; therefore, they could live their life as normal as possible.

#### 4.3.3. Relationship between Set Three Context and Phenomenon: Knowing that I was Not Alone and Living Life above Their Illness

The context—“knowing that I was not alone”—consisted of four contextual features: having a closer relationship, having support from family and friends, and having God watching me. This set of the context showed positive perception of life that the caregivers recognized. The context also represented social and spiritual support available for the caregivers. Even though this kind of support existed, some caregivers did not see it, particularly during the time that they were dwelling in sadness and feeling devastated. Most of the original studies reported and described negative perception of the caregivers of caregiving roles and responsibilities. Only a small number of the studies found positive perception among the caregivers of cancer patients. Positive perception developed among the group of caregivers who had a strong relationship in the family and those who had faith or spiritual strength.

The phenomenon—“living life above the illness”—consisted of three component phenomena: maintaining a meaningful life, accepting support from family and friends, and leaving it in God’s hands. The relationship between this set of the context and its phenomenon showed that positive perception of life led to a better living. In order to lift their life above their illness, the caregivers described that they decided to focus on positive future possibilities. “*Strong reliance*” [61], support from family and friends, and spiritual strength were means to help them to find a meaningful life. However, having support from family and friends did not mean much unless the caregivers accepted the support. Because a caregiving job was demanding, accepting support from family and friends help to reduce the work load. Acceptance is not an immediate response but something that is negotiated and re-negotiated over time [61]. Moreover, for the religious caregivers, their strong faith and belief helped them to overcome a distressed situation because they thought their lives were watched by God. Thus, there was nothing to worry about.

#### 4.3.4. The Relationship between the Three Sets of Contexts and the Caregiving Phenomena

**Figure 2:**
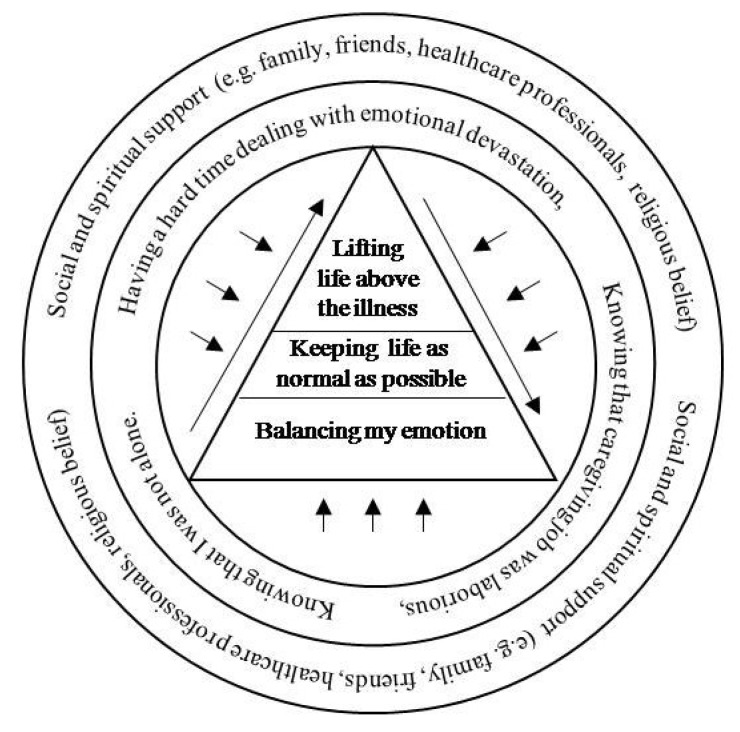
The relationship among the contexts and the phenomena of the experience: the experience of the caregivers who were caring for cancer patients.

Experience is better understood if the context of the experience and the phenomena that structure the experience is well described. The descriptions of each individual context and the caregiving phenomenon as well as the relationship between each set of the context and its relevant phenomenon were described in Section 4.3.1, Section 4.3.2 and Section 4.3.3. After extracting data and categorizing the findings from the original studies, the researchers noticed the relationship among the phenomena. After reading and rereading the original findings many times in order to fill out the phenomena, it became unambiguous that in some cases the phenomenon showed the progress of the caregivers’ thoughts and actions that developed in a better way. The development of the experience was not described elsewhere in the original studies. However, the intuitive analysis of the experience—consciously studying the findings relevant to the caregivers’ actions, intentions, and perceptions—the researchers became more aware of the relationship among each set of the phenomenon and the ongoing progress that the experience was structured. The relationship among the three sets of contexts and the phenomena was illustrated in Figure 2.

The experiences of caregivers caring for cancer patients was illustrated by a model with three phenomena arranged from the general phenomenon to the more specific phenomena: (1) balancing my emotion; (2) keeping life as normal as possible; and (3) lifting life above the illness. Life is dynamic and lived experience. Therefore, caregivers’ perceptions, actions, and intentions changed over time. The changes, particularly the feelings, which were the context of the experience, could be the changes for the better or the worse. A general phenomenon of the experience applied to most of the caregivers who were caring for cancer patients; whereas, a more specific phenomenon was experienced by the lesser number of the caregivers. The most specific phenomenon was the experience that applied only if the caregivers had more positive perception toward caregiving and overcame the negative perception. The phenomena of the experience were surrounded by the contexts that were the world in which the caregivers lived. The way the caregivers perceived their lives influenced their actions and intentions. The surrounding contexts showed in layers. The layer closest to the phenomena was the context that had direct influence to the caregivers’ lives. In contrast, the second layer context further out including social support and spiritual support had either direct or indirect influences to the caregivers’. The second layer of the context showed that the support existed and was available for the caregivers to reach out and get. However, the feasibility of getting and accepting the support depended on the situations and needs of each caregiver.

#### The Context of Caregiving Experience

The context of the experience showed both the positive and negative perception of the situation. Negative perceptions about caregiving were the context that impacted the experience of the caregivers. These perceptions were emotionally draining. The two negative perceptions revealed from data synthesis showed that the caregivers were having a hard time dealing with emotional devastation; meanwhile, they perceived the caregiving job as a laborious job. An intense negative perception reduced a positive mindset and impeded the caregivers’ progress to the better stage of the experience as well as a strong positive perception diminished negative thoughts. The intensity of these feelings was unique to the underlying disease process and treatment or care options, as well as individuals’ backgrounds including social and spiritual supports. This support made available by family, friends, healthcare professionals, and cancer support groups, as well as by spiritual support from prayer groups and religious belief and convictions. During the course of illness, when the negativities were diminished, the caregivers were more conscious of the situation; thus, they could proceed to a higher level of the experience. However, for some caregivers whose patient’s disease continually progressed, social supports were not adequate, or for those who did not have a strong relationship with the patient and family. Therefore, it was hard for them to overcome this emotional burden; consequently they struggled to move to the next stage. However, some studies showed that positive circumstances within negative impacts of being caregivers of cancer patients helped the caregivers to balance their emotions.

#### The Caregiving Phenomena

Three phenomena were arranged from the general phenomenon to the more specific phenomena. The fundamental stage—“balancing my emotion” was a general phenomenon located at the bottom of the pyramid. This phenomenon described the caregivers’ experience when they started caregiving roles. When the caregiving started, the caregivers usually struggled with the truth after learning about the cancer diagnosis. This phenomenon of the experience forms the base of the pyramid, as it represents the fundamental characteristic of the phenomenon as well as showing that it applied to the majority of the caregivers.

The mediated stage—“keeping life as normal as possible”—applied to some of the caregivers. Many caregivers adjusted to the situation and described their experience this way. During the time, the caregivers were caring for the cancer patients, some caregivers were able to overcome the emotional difficulties; whereas, some struggled to adjust to their new role as a caregiver. Therefore at this stage, the caregivers had shifted their focus from the emotional strain and the positive aspect of situation. They were more attentive to the balance of their own needs, as well the needs of the patient. They needed to maintain a normalcy within their life, as well as continued their caregiving role. The positive perception of the situation enabled the caregivers to dealing with the situation more successfully. The caregivers who prevailed over the burden of cancer at this stage were able to respond to the situation more effectively. These caregivers extended more effort to ensure that they spent quality time with their loved one.

The optimum stage—“lifting life above the illness”—showed that the caregivers’ success in adjusting to the caregiving roles. Some caregivers thought positively. For example, after going through the course of the cancer and the treatment, the caregivers realized the cancer could not be cured. In this case, the caregivers shifted their negative thoughts to the good things in lives that they could still find. Some of the caregivers eventually realized that the good things among the worse situation were that they were still together with their loved one. Moreover, they recognized the strength of their relationship with the patients and felt that the cancer and its treatment was something the patients and them went through together. They also perceived that the cancer brought the family closer and that they had family and friends who were willing to support in many ways. These positive perceptions became an inner source of strength building inside the caregivers. This example showed that positive attitude is a foundation of positive acts.

The third and highest stage of the process represents the utmost achievement lived experience. At this stage, the caregivers reconcile the burdens of the situation and can adjust to living with the circumstance of both the cancer and caregiving. This response showed that the caregivers were able to lift their life above the illness. However, only couples and families with strong relationships can develop the ability of coping at this level. During the time the caregivers were caring for the cancer patients, they might encounter unexpected situations due to the effects of cancer and treatments. For the caregivers whose emotions have not been well developed, the situations might bring their thoughts down to the negative point, which affected the response to the situation. Depending on the individual’s background, some caregivers could deal with the situation better than the others.

## 5. Discussion

This present systematic review and meta-synthesis drew together the findings of qualitative research relevant to the experience of cancer patients’ caregivers into a more comprehensible description. Eighteen studies were retrieved from the four major databases, including CINAHL, MEDLINE, Academic Search, and Science Direct, and one Thai database, which was the Thai Library Integrated System (ThaiLIS). After extracting and synthesizing the findings from the original studies, the experiences of the caregivers who were caring for cancer patients were more comprehensible. Thus, this review provided a broader and deeper understanding regarding the experience of the caregivers who were caring for cancer patients.

Following the purpose of the present review, which was to explore the caregiving experience provided for cancer patients, the studies were carefully selected from the databases. The experience of the caregiving at a specific interval of the treatment or a certain stage of cancer was not the focus of this review. Thus, the review did not include studies that focused on exploring the experience of the caregivers caring for cancer patients at a certain stage of the disease, such as at the end of life or at the terminal stage of the disease. The studies that focused on a certain kind of cancer treatment or intervention such as supportive palliative care at the end stage of the cancer were not included either. However, caregiving is an ongoing process. Once cancer is diagnosed, the caregiving role starts and does not cease until the course of the treatment is completed as planned or until the cancer takes the life of the patient. Therefore, the description of the context and the experience as well as the relationship among the contexts and the phenomena presented in this review can be applied to the caregivers caring for the patients at any stage of cancer. Although not every caregiver could move past the burden to the upper level of the phenomena, the fundamental phenomenon is considered an appropriate description of the experience of this group of the caregivers.

Findings from each individual study included in this review described the caregivers’ experience without showing its progression and development. Some studies described only negative experiences and cancer’s impact on caregivers’ lives; whereas, some studies proposed only a positive perception. None of the studies discussed the experience and described the context and the phenomena relevant to the experience. Neither the illustration of the contexts and the relevant phenomena nor the relationship among the two essences of the experiences were displayed and described. Unlike previous systematic reviews, this systematic review adopted Porter’s phenomenological data analysis method [76]. Porter’s method allowed the researchers to capture the essences of the caregivers’ experience that were the context of the experience and the caregiving phenomena. Thus, the findings of this review provide a more comprehensive understanding regarding the caregivers’ experience, particularly the caregivers who were caring for cancer patients.

### 5.1. Description of the Context of the Experience

The illustration of the experience using the pyramid surrounded by the context of the experience shows a close relationship among the direct and indirect socio-physiological influences of the experience, which were the caregivers’ perception of the situation and phenomena that structured their experience. The perceptions were personal and had a direct influence on the way they responded to the situation. Positive perceptions were an inner source of strength. Social and spiritual supports were recognized and the support had an indirect influence on the experience. While the contexts provided more understanding about the caregivers’ lived world, the phenomena provided more understanding around the ways the caregivers lived their lives. This explanation has not yet been provided in literature related to stress and the concept of coping as Kubler-Ross loss [92] and grief coping stages and the concept of the caregiver family caregiving experience (CFCE) [93].

Similar to other studies, the findings of this review showed positive and negative effects of cancer and caregiving. A negative perception such as perceiving that the caregiving job was demanding is both physically and psychologically draining. The perceptions of the caregiving demand consisted of feeling helpless, overwhelmed, inadequate, and uncertain. Perceived emotional devastation including being in shock, being in denial, being in panic, and being fearful was psychologically draining. Negative perceptions in relation to cancer and the burden of caregiving were a hindrance to the development of mature coping.

### 5.2. Description of Caregiving Phenomena

When comparing the caregiving phenomena description presented in this review to the loss and grief coping stages [92] and the concept of the caregiver family caregiving experience (CFCE) [93], it is unarguable that the explanation of the relationship among the contexts and the caregiving phenomena provided a broader and more in-depth explanation regarding the experience. Moreover, the explanation also provided more details about the response to physical and psychological strains. The loss and grief coping stage explains how the person responds to their loss and grief. Denial, anger, bargaining, depression, and acceptance, are the normal reactions to the loss and grief. Each response is a temporary response. The findings from this review also found that the response to the physiological and psychological demands including emotional devastation and a demanding caregiving job was dynamic. However, the response did not necessarily occur in any specific order and often moved between stages before achieving a higher level.

While the CFCE suggests that an ultimate outcome of coping is health and well-being, which includes mental health, physical health, and health-related quality of life [93], the caregiving phenomena stage suggests the development of the experience. The outcome of the experience is the actions and intentions which are the results of the individuals’ perception of their situation. The highest stage of the experience development—“lifting life above the illness”—is the ultimate achievement of caregivers in caring for cancer patients. The CFCE outcomes of coping such as mental health, physical health, and health-related quality of life were measurable and the tools were developed to measure these outcomes. Unlike the CFCE outcomes, the findings of this review emphasized the qualitative information about the caregiving experience that was not measured quantitatively. The only way to examine whether or not the caregivers reached the optimum level of experience development is through their experience that the caregivers share such as the story of their lives as they were living close to the cancer patients.

“Maintaining a meaningful life” was one aspect of “lifting life above the illness”. The results of this study showed that a meaningful life for the caregivers meant being able to enjoy the rest of the time they had with their loved one. Experiencing a sense of closeness with the patient and family members was reported in both qualitative and quantitative studies. However, only a few reports mentioned expression of love and the value of life. Love and passion appeared to be an important means for caregivers to attain a meaningful life. The caregivers who re-evaluated the importance of life discovered a need to spend quality time with their loved one. The results of this review therefore suggested that the devotion of caretakers to their loved one with cancer is a key to finding a meaningful life. When the caregivers found more purpose in their life, they were ready to move on to the next level of the experience.

### 5.3. The Relationship between the Contexts and the Phenomena

Although the description of the phenomenon stage in this study is somewhat similar to the stage of loss and grief coping introduced by [92], the explanation of the context and the phenomena provides more explicit details pertaining to the experience of caregivers caring for cancer patients. The illustration of the experience using the pyramid surrounded by the context of the experience shows a close relationship among the caregivers’ perception and phenomena that structures their experience. The perception is personal and has a direct influence on the way they responded to the situation. Positive perceptions bestow an inner source of strength. Social and spiritual supports are recognized and the support had an indirect influence on the experience. While the contexts provided more understanding about the caregivers’ lived world, the phenomena provided more understanding about the ways the caregivers lived their lives. An alternate comprehensive picture of caregivers’ experience was apprehensible through the explanation of the relationship among the contexts and the phenomena of the caregiving experience.

Other studies reported that the quality of the daily relationship of the caregiver/care-receiver is a central component of the positive aspect of caregiving [94]. Marital adjustment is one of the health and well-being outcomes of the stress process described in the CFCE [93]. Studies that used the Dyadic Adjustment Scale (DAS) showed that there were decreases in marital satisfaction over time among both female and male spousal caregivers [95,96]. In this study, the quality of relationship between caregivers and those for whom they care is a key to successful coping at the highest level whereupon caregivers could live their life above all burdens caused by cancer and care-giving. The caregiver relationship with the patients and family was improved throughout the caregiving process and the relationship was reciprocal between the caregiver and patient [19]. Likewise, this relationship was also found in some of the studies that were included in this systematic review. The results showed that the caregivers and patients go through the caregiving process and developed a relationship called “together-relationship”—a “strong alliance”—that enhanced their abilities to cope with the situation. This relationship helped the caregivers to develop more mature coping; therefore, they could prevail over all the negativity of the situation and look forward to the future. This experience is common in spouse caregivers, but not described by other family members. However, only a few studies reported that the caregivers had achieved this level of the experience.

One important positive perception among caregivers caring for cancer patients was a feeling of accomplishment. The stage of the experiences shows how caregivers develop their ability in dealing with cancer caregiving roles and responsibilities from the fundamental level to the optimum level. At the optimum level, the caregivers felt the most accomplished in their life. This finding is supported by the Conceptual Framework of the Positive Aspect of Caregiving (CAPAC). The conceptual framework contains three main domains of the positive aspects of caregiving: the quality of the daily relationship of the caregiver/care-receiver, a feeling of accomplishment, and the meaning of the role in daily-life. A feeling of accomplishment was reported in the studies that employed a qualitative research method, quantitative research method, and mixed research method. Mixed-method studies reported that the caregivers feel a sense of accomplishment from the knowledge that their care made the patient feel more comfortable, the realization of their own capabilities, a perception of personal satisfaction [97], and from feeling respect and appreciation from their care-receiver [96]. Nevertheless, those studies either conducted in a group of caregivers after the death of the patients or explored the experience of bereavement, which were not included in this review.

## 6. Conclusions

From a systematic review and synthesizing previous studies, the findings of this study provide an expanded knowledge of caregivers’ experiences, particularly caregivers of cancer patients. The experience is revealed as a stage of development. The essences of the experience including perception, intention, and the relationships among these three essences, which have never been described explicitly elsewhere, were explored and described. Thus, this review added more explanation to the area of cancer caregiving. The findings of this review can be used to guide clinical practice and policy formation.

The first recommendation to practice is related to the importance of a positive perspective of caregiving. The first key to successful development is to perceive the positive aspects of the situation. Positive perception helps the caregivers overcome the negative aspects of the situation. Therefore, nurses and healthcare professionals should be more sensitive regarding positive perceptions, aware of their importance, and enhance the development of these perceptions. The more positive the caregivers are about the situation, the better they cope and the higher chance they will accomplish the ultimate goal of living. However, it is challenging to develop a focus in the healthcare of cancer that assists caregivers in finding positive perspectives of being a caregiver.

The second recommendation focuses on preparing the caregivers to assume the caregiver role. The second key to developing successful coping is the sense of accomplishment. The caregivers feel a sense of accomplishment when they see that they can help the patients become more comfortable and able to handle the situation. To enhance these feelings, nurses and healthcare professionals should fulfill the caregiver’s needs by providing them with information, emotional support, and effective medical treatment. The caregivers should also be prepared to deal with the cancer patient’s symptoms and treatment of side effects.

The third recommendation is related to the measurement of the experience. In addition, the differences of the experience at each stage of cancer caregiving should be recognized in order to provide appropriate care and support to the caregivers. The tools to assess the development of the experience and the criteria showing successful development at each stage should be constructed. Appropriate tools will help healthcare professionals better assess the caregiving aspects and enhance caregiver’s abilities to progress to the higher stage of the experience. However, perceptions are sensitive and subjective to each individual and may be difficult to assess. Nurses should develop professional relationships, especially those that build trust and emotional support with the caregivers in order to help caregivers to feel more comfortable and willing to express their feelings.

The fourth recommendation is related to the application of the review study. The healthcare professional that uses the information from this review must be aware of individual differences. The findings of the studies although gathered from reviewing many studies that were relevant to the objectives of the review, showed similarities and differences of experiences. Similarly, experiences were categorized and presented in this study.

The fifth recommendation is for future research. It is critical for nurses and healthcare professionals to have more awareness of these particular stages of coping development and provide appropriate care and support at each stage. Therefore, research on the stages of the experience of caregivers who are caring for cancer patients should be conducted to further the understanding of this concept. Moreover, tools that are more sensitive to measure coping development are needed. To date, no tool has been developed to measure the development of coping. It will be very beneficial for the caregivers if their ability in coping could be assessed more effectively. However, as previous research suggested, an in-depth understating of the caregivers’ experiences could not be achieved only from a quantitative study. A mixed research design of qualitative and quantitative methods may be valuable for further studies of the caretakers’ experience.

## 7. Limitations of the Review

The systematic review used only the results of articles available from the four bibliographic databases and journals available at the university where the researchers are employed and from another university where the second author is doing doctoral studies. However, while the four databases provided comprehensive coverage of key nursing, medical, and health affiliated journals published in English and Thai, some Western and Asian cultural differences, as well as national differences in approaches to health care, were detected while conducting this review. Nevertheless, there were only a few studies in Thai that could be included in this review, and as such there was insufficient information to draw conclusions about the cultural and national differences in caregivers’ experience.

## Abbreviations

CAPAC: Conceptual Framework of the Positive Aspect of Caregiving
CASP: Critical Appraisal Skill Program
DAS: Dyadic Adjustment Scale
CFCE: Concept of the Caregiver Family Caregiving Experience
